# Medical cost and healthcare utilization of amyotrophic lateral sclerosis in China: A cohort study based on hospital data from 2015 to 2018

**DOI:** 10.1097/MD.0000000000023258

**Published:** 2020-11-20

**Authors:** Han Song, Jian-Chao Liu, Zi-Peng Cao, Wen-Jing Luo, Jing-Yuan Chen

**Affiliations:** a Department of Health Service, PLA General Hospital, Beijing; b Department of Occupational and Environmental Health, and the Ministry-of-Education's Key Laboratory of Hazard Assessment and Control in Special Operational Environment, School of Public Health, Fourth Military Medical University, Xi’an, China.

**Keywords:** amyotrophic lateral sclerosis, healthcare utilization, medical big data, medical burden, neuroepidemiology

## Abstract

Amyotrophic lateral sclerosis (ALS), a specific neurodegenerative disease, imposed increased economic and utilizations burden on the healthcare system, especially with the progress of the diseases severity. However, the economic burden on Chinese ALS patients remained unclear. This study therefore was aimed to investigate medical cost and healthcare utilization for Chinese ALS patients.

Longitudinal health data of over 20 million individuals, including military personnel and civilians, was collected from all Chinese military hospitals. We identified 480 patients with a first major diagnosis for ALS from 2015 to 2018, while matched 400 controlled patients on age, gender, ethnic group, geographic region, length of stay, year of diagnosis and comorbidity. Their medical cost and healthcare utilizations were then measured 1 year before, and 1 year after ALS diagnosis.

The median annual medical cost of ALS patients was about 2-fold higher, 17,087 CNY during the index year than 1 year before, 7859 CNY. The highest increase in utilizations may account for medical costs on ALS patients, which was represented by hospitalizations (Odd Ratio (OR) = 4.26, 95% confidence interval (CI) 3.52, 5.15), electromyography (OR = 4.14, 95% CI 2.37, 7.22), nerve conduction velocity (OR = 3.26, 95% CI 2.23, 4.77).

This study is the first one reporting direct economic burden on Chinese ALS patients. Efforts should be made to develop cost-effective diagnostic tools in order that sources of medical cost were more effectively allocated, and this disease was detected earlier.

## 1 Introduction

Amyotrophic lateral sclerosis (ALS), also known as a motor neuron disease or Lou Gehrig disease, is a specific neurodegenerative disease that progressively causes the death of upper and lower motor neurons controlling voluntary muscles. To our knowledge, ALS is an incurable disease, characterized by stiff muscles, muscle twitching, and gradual worsening of weakness. ALS had a poor prognosis and high mortality with patients died within 3 to 5 years due to respiratory paralysis.^[1]^ The incidence of ALS was estimated to be 1.9/100,000/yr worldwide,^[2]^ whereas in China, the annual incidence or prevalence of ALS is lower than developed countries, such as Japan, Europe, or Australia.^[3]^

It is estimated that number of ALS patients will increase 69% from 2015 to 2040 across the world.^[4]^ ALS is relatively rare, yet imposing considerable economic and social burden. A recent study reported that its annual total cost per patient ranged from US$ 13,667 in Denmark to as high as US$ 69,475 in the United States.^[5]^ Another study in the USA found monthly costs per patient increased 9 months before diagnosis, peaked at the index month (Medicare: US $10,398; commercial: US $9354) and decreased but remained high post-index.^[6]^ Diagnosis of ALS is primarily based on the symptoms and signs after ruling out other diseases. In the United States, $10,000 to $20,000 per patient was needed for a neurologist to get a confirmative ALS diagnosis.^[7]^ However, economic burden on Chinese ALS patients has not been reported. Phenotypic differences, like mean age-at-onset or diagnostic delay, were found among German and Chinese ALS patients.^[8]^ In addition, clinical characteristics and outcomes of Chinese patients with sporadic ALS were quite different compared with patients from other countries.^[9]^ These findings may indicate the sources of differences in economic burden between Chinese ALS patients and other groups.

Further, cardiovascular, metabolic, or neuropsychiatric comorbidities of ALS, make it difficult to isolate the specific contribution of ALS to medical economic burden.^[5]^ Thus, we aimed to investigate medical cost and healthcare utilization for Chinese patients with ALS in the study where the control group had similar comorbidities, and under the correspondingly the same medical insurance system [Urban Employees (UE), Urban and Rural Residents (URR), Free Medical Care (FMC), and Self-Pay Medical Care (SPMC)]. The data set, derived from all military hospitals in China, provided an edge in studying rare disease, like ALS, as it includes over 20 million people, representing a possibly largest scale of Chinese ALS patients of all ages. The estimated medical cost and healthcare utilization will provide insight into offering reasonable amount of financial support for Chinese ALS patients and will improve allocation and efficient use of medical resources.

## 2 Methods

### 2.1 Data resource

A retrospective analysis was done with medical big data collected from all Chinese military hospitals, which serve not only the military personnel, but also civilians, taking up a significantly high proportion of Chinese health services.^[10]^ Hospitals in this study are mostly located in key cities of provinces. The database includes patients’ demographic information, vital signs, diagnoses of diseases, medical orders, examination reports, lab tests results, drugs, medical supplies, or even infections. The present study had analysis performed with historical de-identified data; thus, it was exempt from Institutional Review Boards (IRB) Committee of Fourth Military Medical University.

### 2.2 Study population

Study population comprises patients with a first major diagnosis for ALS by referring to the International Statistical Classification of Diseases, and Related Health Problems (ICD-10) G12.2 (motor neuron disease). We excluded subjects diagnosed with other systemic atrophies including G10 (Huntington disease), G11 (Hereditary ataxia), G13 (Systemic atrophies primarily affecting central nervous system in diseases classified elsewhere). The Day 1 for hospitalization of ALS patients was defined as the index date.

**Figure 1:**
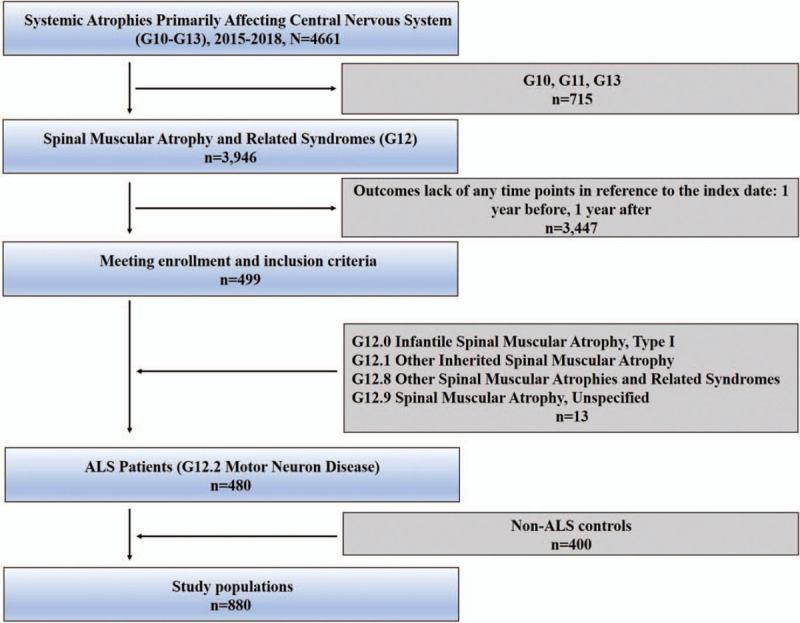
Flow diagram of patient selection process. G10, Huntington Disease. G11, Hereditary Ataxia. G13, Systemic atrophies primarily affecting central nervous system in diseases classified elsewhere.

A total number of 880 inpatients of all military hospitals from 2015 to 2018 were included as subjects by nonprobability sampling using structured query language expression of Oracle databases. Records lacking in any primary keys, including hospital information, patient's identity, and visit times, were excluded. The control groups were set with criteria that patients should have at least one hospitalization visit and no diagnosis code for ALS or other nervous system diseases. The controls were match on age, gender, ethnic group, geographic region, length of stay, year of diagnosis and comorbidity. Figure 1 illustrates the detailed scheme.

### 2.3 Covariates and outcomes of interest

The ALS subjects and controls were matched on these comorbidities to maximize the association of medical cost and health care utilization with ALS. The structures of medical cost, outpatient visits, hospitalizations, emergency department visits, laboratory tests, imaging [Electromyography (EMG), nerve conduction velocity (NCV), Ultrasound, Computed Tomography, and Magnetic Resonance Imaging (MRI)] were selected as outcomes of interest. We measured the outcomes at 2 time points in reference to the index date: 1 year before, 1 year after.

### 2.4 Statistical analysis

Univariate descriptive analysis was employed to described quantitative and qualitative variables as count or median. A collection of summarization techniques has been formulated as bubble chart, scatter plot by groups, scatter plot on maps. We compared outcomes of interest between ALS patients and controls on 1 year before, 1 year after their diagnosis date. Medical cost structure was analyzed based on all details of medical expenses. Healthcare utilization was identified as events and Odd Ratio (OR) (events 1 year after ALS diagnosis/controls/ events 1 year before ALS diagnosis/controls). Without using samples to infer the population, we showed current status of Chinese ALS patients through statistical description. Data were analyzed with SPSS version 24.0, illustrated using GraphPad Prism 6, and Python (version 5.1.0; Anaconda3).

## 3 Results

We identified 3946 people with a first diagnosis of Spinal Muscular Atrophy and Related Syndromes (G12) from 2015 to 2018, of which 3447 were excluded for lack of medical records 1 year before and 1 years after the index diagnosis. The remained 480 people with ALS (G12.2) and 400 non-ALS patients, hitting a total of 880 people (Fig. 1) as the study population, were matched by age, gender, ethnic group, geographic region, length of stay, year of diagnosis.

### 3.1 Demographic characteristics

The mean ± SD (range) age of ALS patients in our study was 57.37 ± 15.09 (7–92) years old at the index date. Male or female patients accounted for 61.88% or 38.12%, respectively. We depicted demographic factors including gender, age, ethnic group, marital status, preadmission conditions, blood type (ABO and Rh), insurance type among ALS patients and matched controls (Table 1). Interestingly, in terms of ABO blood type, type-O took the highest proportion, 20.21% in ALS groups, in contrast to a mere 7.00% in control groups. As rare blood types, proportions of Rh-negative in ALS patients (10.63%) were far higher than that of the controlled cases (0.50%).

**Table 1:** Characteristics of ALS Patients and matched controls.

Categorical	Variables	ALS (n = 480)	Controls (n = 400)
Gender	Male	297	203
	Female	183	197
Age	<18	1	–
	18–45	81	19
	45–60	169	57
	>60	229	324
Ethnic group	Han	467	400
	Minority	13	–
Marital status	Married	448	391
	Unmarried	27	1
	Divorced	3	1
	Widowed	2	7
Preadmission conditions	Normal	443	348
	Urgent	37	49
	Dangerous	–	3
Blood type (ABO)	A	51	35
	B	49	17
	O	97	28
	AB	10	9
	Untested	273	311
Blood type (Rh)	Negative	51	2
	Positive	104	61
	Untested	325	337
Insurance Type	URR	80	17
	UE	215	319
	FMC	77	9
	SPMC	108	55

ALS = Amyotrophic Lateral Sclerosis, FMC = Free Medical Care, SPMC = Self-Pay Medical Care, UE = Urban Employees, URR = Urban and Rural Residents.

### 3.2 Medical costs in ALS

**Figure 2:**
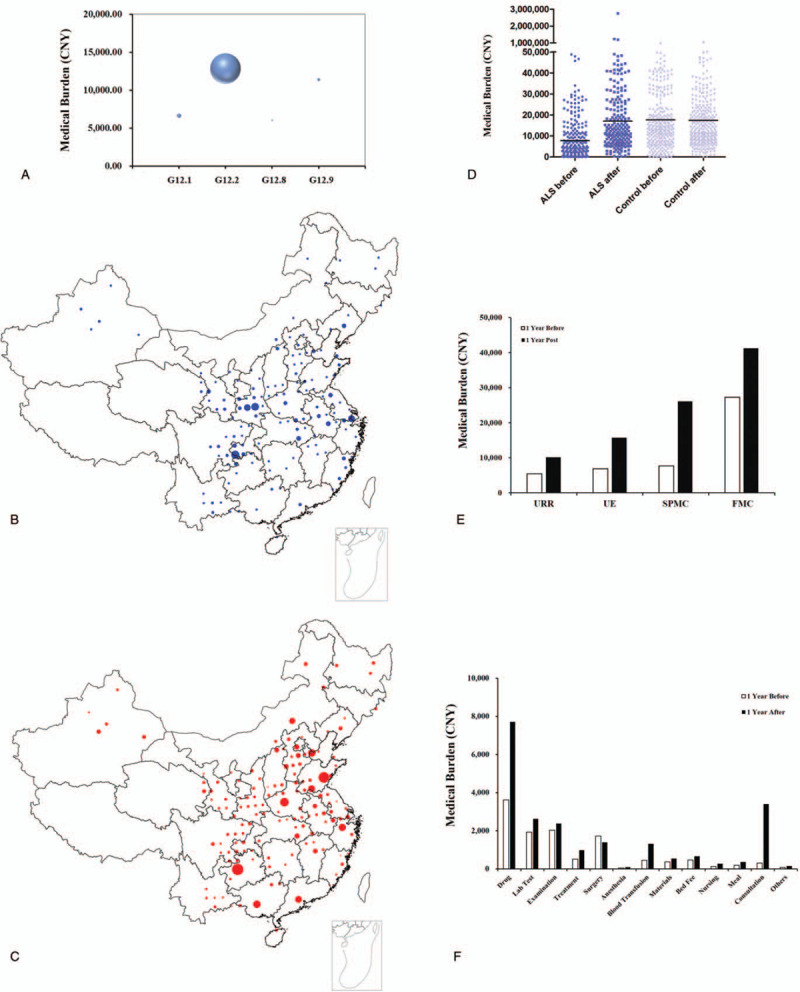
Medical burden of ALS or controlled patients. (A) Bubble charts of personal medical burden of subcategories within G12. (B, C) Detailed geographical distribution of ALS patients. Size of the symbols reflects the number of individuals. (B), and medical costs (C). (D) Scatter plot of Medical burden between ALS or controlled patients within peri-diagnosis period (1 year before vs 1-year post). The line within scatters indicates median of medical cost. (E) Median of annual medical cost among ALS patients with four system UE, URR, FMC, or SPMC 1 year before or 1-year post. (F) The structures of medical cost for ALS patients 1 year before or 1-year post.

Within the specific three-character category G12, we compared the number of patients and corresponding medical cost between its subcategories, where G12.2 (motor neuron disease) took the largest proportion of the medical expenses, 12,852 CNY (Fig. 2A). Further, we carried out an analysis on geographical characteristics of ALS patients via their birthplace codes in county areas in China, which showed that major ALS patients came from Shaanxi (65), Chongqing (44), Hebei (37), Shandong (28), Jiangsu (23), Sichuan (21) (Fig. 2B). As for medical cost per patient, Guizhou, Guangdong, Henan reported higher medical cost than the rest provinces and regions, with a total of 117,665 CNY, 85,040 CNY, and 69,281 CNY, respectively (Fig. 2C).

Figure 2D shows the annual total medical costs per patient among ALS patients and controlled patients during peri-diagnosis period (1 year before vs 1 year post). The median cost of ALS patients was about 2-fold higher 17,087 CNY [Interquartile Range (IQR) 8623 CNY – 41,584 CNY), during the index year than 1 year before, 7859 CNY (IQR 2477 CNY – 25,703 CNY). We further investigated the annual cost of following medical insurance system UE, URR, FMC, or SPMC. Specifically, the median cost of medical care increased highest at SPMC groups, by 3.41-fold during the year following ALS diagnosis, from 7633 CNY (IQR 2646 CNY – 19,291 CNY) to 26,083 CNY (IQR 7232 CNY – 63,241 CNY) the year after diagnosis. The average cost for UE, URR or FMC increased by 2.29, 1.86, or 1.51-fold, respectively (Fig. 2E). For reference, the annual healthcare cost of non-ALS controls was almost constant between 1 year before (17,676 CNY (IQR 9518 CNY – 42,856 CNY)) and 1 year post 17,498 CNY (IQR 8909 CNY – 38,495 CNY) (Fig. 2D). We further analyzed the structure of medical cost for ALS patients, where drug cost took up a large proportion of the medical expenses, with median of drug cost climbing from 3615 CNY (IQR 929 CNY – 10,410 CNY) to 7709 CNY (IQR 3098 CNY – 22,207 CNY) during the year after diagnosis. In comparison, the consultation fee increased, about 11-fold, from 305 CNY (IQR 188 CNY – 623 CNY) to 3400 CNY (IQR 1700 CNY – 6600 CNY) (Fig. 2F), highest among all the factors.

### 3.3 Healthcare utilization in ALS

On the basis of estimating medical cost, we further determined healthcare utilization parameters at the index date and 1 year before, respectively. Compared with the year before the index date, utilization parameters, like hospitalizations, EMG, NCV, MRI, increased markedly during the following year among ALS patients, which were not observed in controlled group. The largest increase in utilization (cumulative events) for ALS patients was hospitalizations, 4.67-fold, followed by EMG, 4-fold, NCV, 2.77-fold. The OR and 95% confidence interval for hospitalizations, EMG, NCV, or MRI were 4.26 (3.52, 5.15), 4.14 (2.37, 7.22), 3.26 (2.23, 4.77), or 2.29 (1.77, 2.95), respectively. However, imaging tests and ultrasound showed minimal increase (OR = 0.83, 95% confidence interval 0.68, 1.00) (Table 2).

**Table 2:** Cumulative events for Patients within peri-diagnosis period.

	ALS before	ALS after	Control before	Control after	OR (CI)
n		480		400	
Hospitalizations	157	733	1147	1258	4.26 (3.52, 5.15)
Outpatient visits	1444	2273	1715	1552	1.74 (1.58, 1.91)
ED visits	169	392	109	255	0.99 (0.74, 1.32)
Laboratory tests	4025	9973	6934	7604	2.26 (2.15, 2.37)
EMG	97	388	30	29	4.14 (2.37, 7.22)
NCV	113	313	86	73	3.26 (2.23, 4.77)
Ultrasound	368	681	283	635	0.83 (0.68, 1.00)
CT	128	225	134	175	1.35 (0.99, 1.84)
MRI	206	455	205	198	2.29 (1.77, 2.95)

ALS = Amyotrophic Lateral Sclerosis, CI = Confidence Interval, CT = Computed Tomography, ED = Emergency Department, EMG = Electromyography, MRI = Magnetic Resonance Imaging, NCV = nerve conduction velocity, OR = Odd Ratio.

**Figure 3:**
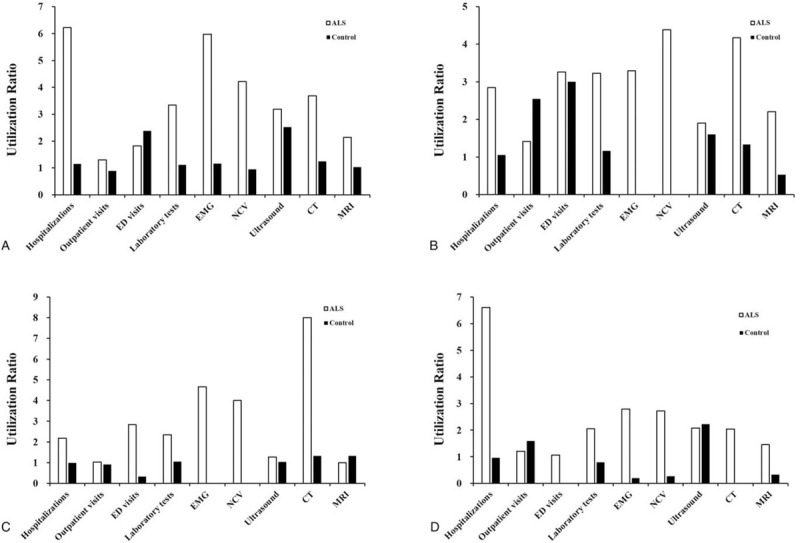
Healthcare utilizations among ALS or controlled patients. The bars represent utilization ratio of utilization parameters (cumulative events 1 year after ALS diagnosis/controls/events 1 year before ALS diagnosis/controls) in patients with following systems, UE (A), URR (B), FMC, (C) and SPMC (D).

Figure 3 demonstrates the utilization ratio among URR, UE, FMC, or SPMC patients with ALS compared with matched controls. Patients experienced large increase of hospitalizations for UE (6.23-fold) and SPMC (6.60-fold) patients, but relatively mild increase for URR (2.84-fold), FMC (2.18-fold) 1 year after ALS diagnosis when compared with 1 year before indexed date. As vital ALS diagnosis tools, URR, UE, FMC or SPMC patients with ALS reported 3.29, 5.97, 4.67, or 2.79-fold increase of EMG tests, 4.38, 4.22, 4.00, or 2.72 increase of NCV tests, respectively.

## 4 Discussion

Investigation on the data retrieved from a large military medical database show that medical costs for ALS patients were close to that of the non-ALS control group under similar conditions. However, the total annual cost increased about 2-fold during the ALS diagnosis year, far higher than the controls, among which medical costs during the ALS diagnosis year reached 10,170 CNY, 15,734 CNY, 41,253 CNY, 26,083 CNY with URR, UE, FMC, or SPMC, respectively. As major sources of medical costs, drug usage took a high proportion of economic burden for ALS patients. As the healthcare utilization encompassed hospitalizations, laboratory tests, EMG, or NCV test, of its increment may, to some extent, explain the increase of medical costs.

The onset of ALS is insidious and to our knowledge, it is difficult to get definite diagnosis with single diagnostic tool, therefore physicians always determined the ALS based on symptoms and signs.^[11]^ Differential diagnosis were needed to rule out diseases, such as multifocal motor neuropathy or multiple sclerosis, whose symptoms closely resemble ALS.^[12]^ Compared to the 11 months (range: 6–21) diagnostic delay from ALS onset in a cohort of 260 patients in Italy,^[13]^ median diagnostic delay in China was 14 months,^[9]^ indicating a relatively high medical costs during outpatient visits or hospitalizations in the extended process for ALS diagnosis indicated.

The mean age or male to female ratio was consistent with previous results. The mean age at onset was 52.4 years at 10 ALS centers of Chinese ALS Association from March 1, 2009 to August 31, 2009.^[14]^ The male to female ratio was 1.69:1 among 2101 ALS patients collected in the University Third Hospital, Beijing, China.^[15]^ To our knowledge, practical diagnostic biomarkers in the blood or cerebrospinal fluid has not been developed for the ALS disease.^[16]^ As rare blood type, the proportion of Rh-negative patients in ALS groups were about 20-fold compared with the matched controls. In previous report, no association of specific blood groups was found between ALS and controls.^[17]^ The differences may be derived from the study population, or the study design. Though there is no proper account for this high constituent ratio of type-O or Rh-negative type yet, these findings may contribute potentially to the early detection of ALS in Chinese patients.

As a relatively rare disease, the most cost-effective modality to predict the severity of ALS has not been clearly illustrated. In this study, ALS treatment was examined by referring to existing medical practices, like EMG, NCV, or MRI test. To be specific, EMG, as key differential diagnosis tools, was used to detect electrical activity in muscles, identifying loss of lower motor neurons;^[18]^ EMG abnormalities were observed in approximately 40% of asymptomatic limb muscles in 150 Chinese ALS patients.^[19]^ NCV, another diagnostic tool to assess peripheral nerve conduction function, were used to exclude the possibility of peripheral neuropathy or myopathy.^[20]^ The computed tomography and ultrasound tests increased slightly during ALS diagnosis compared with controls, implying that some examinations may have been used unnecessarily. Thus, the medical cost and corresponding utilizations during the first diagnosis of ALS suggested that more efficient tools of screening and assessment were needed.

This study also bridged some gaps in the existing literature. First, until our study, no direct economic burden for Chinese ALS patients from a large medical database was reported. Unlike in USA,^[21]^ Ireland,^[22]^ and Canada,^[23]^ annual medical costs per Chinese ALS patient with 4 system, URR, UE, FMC, or SPMC were far lower than western developed countries, reflecting the gap of economic level. Second, by establishing matched controls with similar conditions as references, we eliminated confounding factors driven by other complications. Third, we retrieved medical record during outpatient visits or hospitalizations before the first ALS diagnosis and made it possible to analyze variances of cost and healthcare utilizations brought by this disease.

This study has some limitations. There may exist population selection bias, for patients with ALS who once visited civilian hospitals, are not captured in this medical database. Besides, economic burden outside hospitals, such as assistive devices or caregiver burden, has not been calculated into the overall medical burden of ALS. Third, we were not able to distinguish among appropriate and excessive use of medical resource because of the inherent limitation of hospital-derived databases.

## 5 Conclusions

In summary, this study reported current status of economic burden for Chinese ALS patients. Currently, no effective cures for ALS or treatments were found to stop disease progression. From perspective of medical big data development, efforts should be made to develop cost-effective diagnostic tools so that this disease was detected earlier and economic burden was reduced for each ALS patient.

## Acknowledgments

We thank Qing-Yi, Wang for English improving.

## Author contributions

**Investigation:** Han Song, Wenjing Luo, Jingyuan Chen.

**Methodology:** Han Song, Jianchao Liu, Wenjing Luo, Jingyuan Chen.

**Project administration:** Jingyuan Chen.

**Resources:** Jingyuan Chen.

**Software:** Jianchao Liu.

**Validation:** Jianchao Liu, Wenjing Luo, Jingyuan Chen.

**Visualization:** Zipeng Cao.

**Writing – original draft:** Han Song, Jingyuan Chen.

**Writing – review & editing:** Zipeng Cao, Wenjing Luo, Jingyuan Chen.
